# Beliefs, Behaviors, and Practices of Farm Biosecurity in the Midwestern U.S. Swine Operations

**DOI:** 10.3390/ani15172515

**Published:** 2025-08-27

**Authors:** Maurine C. Chepkwony, Colin Yoder, Marie R. Culhane, Maria Sol Perez Aguirreburualde, Andres M. Perez, Cesar A. Corzo, Dennis N. Makau, Michael W. Mahero

**Affiliations:** 1 Center for Animal Health and Food Safety, University of Minnesota, Minneapolis, MN 55108, USA; cmcherotich@gmail.com (M.C.C.); yoder065@umn.edu (C.Y.); mperezag@umn.edu (M.S.P.A.); aperez@umn.edu (A.M.P.); 2 Department of Veterinary Population Medicine, College of Veterinary Medicine, University of Minnesota, Minneapolis, MN 55108, USA; grame003@umn.edu (M.R.C.); corzo@umn.edu (C.A.C.); 3 Department of Biomedical and Diagnostic Sciences, College of Veterinary Medicine, University of Tennessee, Knoxville, TN 37996, USA

**Keywords:** swine biosecurity, risk perception, producer behavior, secure pork supply (SPS), foreign animal disease preparedness

## Abstract

Effective biosecurity is crucial for preventing swine diseases, but there can be a gap between what producers intend and what actually happens on the farm. We conducted a cross-sectional, self-administered online survey of Midwestern US swine operations (N = 54) to explore beliefs and practices related to the Secure Pork Supply (SPS) plan. Because some questions had item-level nonresponse, we report proportions for the full sample (denominator = 54) and, where helpful, for those who responded to specific items (sample size varies). Across the full sample, 27.8% (15/54) said they use biosecurity measures always or sometimes. For enhanced biosecurity, 24.1% (13/54) marked always and 3.7% (2/54) sometimes—with all item responders (N = 15) indicating some level of use. Among those who answered, the influence of veterinarians was clear: 81.8% (9/11) said their vet’s opinion is always important when deciding on biosecurity (compared to 16.7% in the full sample). Confidence in controlling an outbreak was similarly high among those who responded: 92.9% (13/14) reported they could always or sometimes control spread (24.1% in the full sample). That said, uptake of several specific SPS components was low—for example, only 27.3% (3/11) reported always monitoring or recording crossings of the line of separation, while 63.6% (7/11) said never. No single biosecurity practice was universally adopted, and overall, fully SPS-aligned programs appeared uncommon in this Midwestern group. These descriptive findings point to clear targets for improvement—such as better monitoring of the line of separation—and highlight potential leverage points, especially the role of attending veterinarians, for outreach efforts. Because this was a convenience sample relying on self-report and had item nonresponse, the results are not statistically representative of the broader regional industry but do provide valuable insights into respondent practices.

## Simple Summary

Preventing disease on pig farms depends heavily on good biosecurity practices, but there is often a gap between what producers believe they are doing and what actually happens on the ground. In this study, we surveyed swine operations in the Midwest to understand how they think about and implement biosecurity. Most respondents said they value biosecurity and feel confident managing disease risks, but fully Secure Pork Supply (SPS)-aligned programs were uncommon. Some practices—such as tracking movement between clean and dirty areas—were commonly overlooked, despite their importance in controlling outbreaks. Among majority respondents, veterinarians strongly influence management decisions, which suggests that veterinarians could play a bigger role in supporting on-farm biosecurity. While this was a relatively small study, the findings offer useful insight into how beliefs and practices align—and where they do not. Helping producers build a clearer understanding of what enhanced biosecurity means could go a long way toward strengthening preparedness, especially with ongoing global threats such as African swine fever.

## 1. Introduction

The US swine industry is one of the largest pork contributors to the global pork value chain, contributing approximately 12% of the world’s pork, estimated at more than 8 billion USD in export value [1,2]. As such, the swine industry is economically crucial in the US and globally. Due to its vertical integration [3] the industry is often troubled by various endemic viral and bacterial diseases resulting in huge financial losses—more than USD 600 million per year from porcine reproductive and respiratory syndrome (PRRSV) [4]. Although the swine industry appears to have mastered how to mitigate the impacts of most of these swine diseases through vaccination and application of different biosecurity measures, the ever-present threat of introduction of foreign animal diseases (FADs) such as African Swine Fever (ASF) [5] necessitates regular evaluation and improvement of these biosecurity measures. Previous studies have highlighted some inconsistencies and complexities in the adoption of biosecurity recommendations such as those described in the secure pork supply (SPS) plan [6,7]. As part of a larger study aimed at understanding swine biosecurity management in the US swine industry [8], this study sought to characterize the beliefs, behaviors and practices regarding the adoption and implementation of biosecurity measures by midwestern US swine production systems on their farms.

## 2. Materials and Methods

Although this online survey study was intended for all US swine production enterprises, the responding production enterprises were exclusively from the Midwest region. We recorded feedback from 122 pig producers in the Midwest US between December 2021 and September 2022 using Qualtrics. We used an anonymized self-administered online questionnaire to collect data for our study (Supplementary Material Table S1). The questionnaire was disseminated using a link emailed through outreach networks and databases, pork council and associations such as the American Association for Swine Veterinarians. The questionnaire was developed borrowing tools from similar studies [7,9,10]. We subsequently pretested the questionnaire among 10 veterinarians, extension personnel and producers and used their feedback to update and refine the questions before deployment for data collection. The data captured details on experience of the respondents in swine production, perception of FAD risk/threat and behavioral, normative and control beliefs regarding biosecurity practices as prescribed in the secure pork supply (SPS) plan [6]. Respondent job role (such as owner/operator, manager, veterinarian) was not collected; therefore, we refer to all participants as ‘respondents’. All data were handled in accordance with recommended data privacy and protection guidelines. Descriptive statistical analysis using R 4.2.0 software [11] including frequency summary statistics were done to identify underlying patterns of biosecurity practices and beliefs among respondents. We summarize the terminology used in the questionnaire in Box 1, distinguishing baseline biosecurity from SPS-aligned enhanced biosecurity and listing the specific components assessed. Generative artificial intelligence (GenAI) tools were not used in the design, data collection, analysis, interpretation, or generation of any content in this study.

Box 1 **Operational definitions and components of Biosecurity and Enhanced Biosecurity (SPS Plan) [7,12].**

Biosecurity (general):

- Structural biosecurity: Physical elements such as facility design and maintenance aimed at preventing disease entry.
- Operational biosecurity: Management practices preventing disease introduction and spread (e.g., cleaning and disinfection, movement controls).
- Key concepts include:
  ○ Cleaning and Disinfection (C&D) stations
  ○ Use of personal protective equipment
  ○ Monitoring animal and vehicle movements

Enhanced Biosecurity (SPS Plan):

- A written, site-specific enhanced biosecurity plan developed and managed by a designated Biosecurity Manager.
- Defined Perimeter Buffer Area (PBA) surrounding the production site to reduce risk from adjacent areas.
- Defined Line of Separation (LOS) within the site separating clean and potentially contaminated zones with controlled access points.
- Regular biosecurity training and documentation of personnel.
- Premises map detailing key biosecurity infrastructure and pathways (site entry, PBA, LOS, C&D stations, parking, carcass disposal, vehicle routes).
- Contingency planning for foreign animal disease (FAD) events and disease introductions.

## 3. Results and Discussion

This study provides a first, concise snapshot of SPS-aligned beliefs, behaviors, and practices among midwestern swine operations, quantifying specific gaps. Considering unique link clicks (N = 122) as the denominator, the completion rate among link clickers was 44.3% (54/122). Because we did not recruit from an enumerated sampling frame with known inclusion-probabilities, this completion figure should not be interpreted as a population response rate.

### 3.1. Respondent Characteristics

The completion rate among link clickers was 44.3% (54/122) considering the number of individuals who clicked on the survey link (N = 122) as the total. The total number of responses was 54 and thus subsequent analysis was based on N = 54 as the sample population. Noncompletion in an open, voluntary survey should not be seen as indifference. Producers often face time and labor pressures, survey fatigue, and may hesitate to share sensitive biosecurity information without strong confidentiality guarantees. Whether they think the survey is relevant and when it is conducted—like during busy seasons—also plays a role. Since we did not collect data on why people did not participate, we avoid guessing their reasons.

The mean age of the respondents was 44.1 ± 12.9 years, with a median of 44 years and ranging from 15–64 years. These respondents had also been in swine production for 23.0 ± 14.8 years, with a median of 20 years and ranging from 2–58 years. A total of 22.2% (12/54) of the respondents had college level education, and about 5% of the respondents had high school level education. About a quarter (25.9%, 14/54) of the respondents to the questionnaire were independent producers and about 5.6% (3/54) were strictly finisher farms. Most, 11.1% (6/54) of the responding operations had an inventory of >2000 pigs and 9.3% (5/54) had an inventory of more than 5000 pigs. These distributions describe our midwestern respondents and reflect several operation types and sizes present in the region; however, given the modest sample size, the findings should be interpreted as descriptive of respondents rather than statistically representative of the regional industry.

### 3.2. Behaviors

Among the respondents who answered questions on their perceptions and practices of implementing biosecurity measures (Box 1) various factors influenced their actions and response to endemic or foreign animal diseases. Overall, the operations that responded reported they always (22.2%, 12/54) or sometimes (5.6%, 3/54) implemented biosecurity measures. Fewer than half chose “always” for many practices—which was not unexpected. “Always” sets a high bar; plenty of producers adhere to routine biosecurity practices, but not in every situation (think after-hours loadouts, unexpected service calls, or work off-site). Resource and infrastructure limitations—such as staffing, time, facility layout, or access to cleaning and disinfection—make “always” difficult to sustain, especially for smaller or independent farms, which made up much of our sample. Context also matters: finisher-only vs. farrow-to-finish, single-site vs. multi-site, and how much risk producers actually perceive if disease pressure is low. Finally, how respondents interpreted “always” and the nature of self-reporting probably pushed responses away from the absolute. For this survey, we did not ask about reasons, so these inferences are based on our sample and everyday production realities.

### 3.3. Beliefs

**Figure 1:**
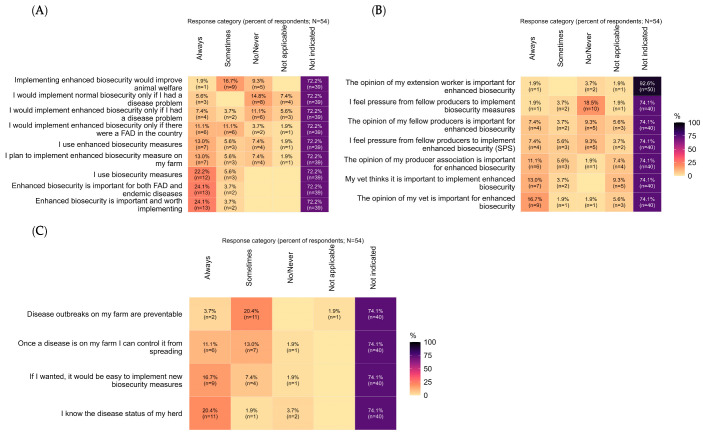
Summary of behavioral, normative, and control beliefs about swine biosecurity among midwestern swine operations. (**A**) Behavioral beliefs; (**B**) Normative beliefs; (**C**) Control beliefs. Rows are statements; columns are response categories (Always, Sometimes, No/Never, Not applicable, Not indicated). Cells display % of respondents (based on page completes, N = 44) and (n); rows are sorted by % Always within each panel. Not indicated shows item-level nonresponse. Color scale represents percentage (darker = higher). Full counts and item-level Ns are provided in Supplementary Table S1. Sample: respondents from Midwestern U.S. operations; estimates are descriptive and not statistically representative. Definitions of “biosecurity” and “enhanced biosecurity (SPS-aligned)” appear in Box 1.

Most responding operations believed that enhanced biosecurity was always (24.1%, 13/54) important and worth implementing or at least sometimes (3.7%, 2/54). In equal measure, respondents felt that enhanced biosecurity was important for both FAD and endemic diseases (always: 24.1%, 13/54; sometimes: 3.7%, 2/54). Additionally, 22.2% (12/54) indicated they would use enhanced biosecurity if there were a threat of FAD in the country (Figure 1A).

Some of the primary biosecurity practices where we observed discordance were the presence and use of a functional line of separation (LOS) to distinguish between clean and dirty areas, and whether movements across the LOS were monitored/recorded. Nonresponse on LOS items was high—74.1% (40/54) did not indicate whether they had an LOS, and 77.8% (42/54) did not indicate whether it was defined/functional—suggesting possible sensitivity or definitional ambiguity. However, among item respondents, about nine in ten reported observing the LOS appropriately—for example, restricted access across the LOS (90.9%, 10/11) and sufficient LOS size (90.9%, 10/11). Despite this, more than half (63.6%, 7/11 of respondents to that item) never monitored or recorded movements across the LOS. Good biosecurity practice recommends tracking LOS crossings to identify potential breaches or contamination routes, especially during outbreaks [7,8]. More awareness on how to effectively use LOS may be beneficial to the industry for improved disease management.

### 3.4. Practices

Respondents’ actions to implement or update enhanced biosecurity measures were influenced by various sources, with attending veterinarians and the swine producers’ association being the most frequently endorsed influencers. Although the full sample showed variable responses, veterinary influence was clearly predominant among those who responded to the items. For example, 13.0% (7/54) of respondents reported that their veterinarian always emphasizes the importance of enhanced biosecurity, and among item responders, 77.8% (7/9) indicated always. Similarly, 16.7% (9/54) marked that their vet’s opinion is always important for implementation decisions, with 81.8% (9/11) of item responders affirming this. Producer-association influence was less frequent but still notable, with 11.1% (6/54) of the full sample marking always, and 60.0% (6/10) of item responders endorsing this. In contrast, peer pressure appeared minimal; 76.9% (10/13) of item responders reported no/never feeling pressure from fellow producers to implement biosecurity measures. Regarding contingency use, 22.2% (12/54) indicated they would adopt enhanced biosecurity only in response to a national foreign animal disease (FAD) threat (Figure 1B).

While a substantial proportion of respondents did not select “always” at the whole-sample level, likely reflecting item nonresponse, confidence among respondents was generally high. For instance, 20.4% (11/54) indicated they always know the disease status of their herd, with 78.6% (11/14) of item responders affirming this. Regarding outbreak preventability, respondents predominantly considered outbreaks sometimes preventable (84.6% of item responders) rather than always. When faced with an outbreak, most respondents believed they could control spread either always or sometimes (92.9% of item responders) (Figure 1C).

Despite some implementation of enhanced biosecurity elements, item-level data reveal gaps across multiple SPS components. These included the presence of a biosecurity manager, a functional perimeter buffer area (PBA), designated vehicle and equipment cleaning and disinfection points, active lines of separation with restricted crossings (LOS), policies on equipment sharing, controlled building access (locks), and protocols for receiving supplies and feed; these align with SPS recommendations [6]. Both external and internal biosecurity are critical for preventing disease introduction and spread [13,14]. This is especially true for pathogens such as PEDV, PRRS, and influenza viruses—where fomite/mechanical transmission routes are plausible—and vehicle hygiene plays a key role [7,15,16,17,18]. Likewise, maintaining a clear LOS and adhering to hygiene protocols (e.g., protective clothing, shower-in/shower-out) reduces spread and supports biocontainment [19,20]. Our findings indicate that no single practice was universally adopted, and comprehensive SPS-aligned biosecurity programs were uncommon among respondents.

### 3.5. Sources of Influence and Considerations

While there are varied reasons why farms may respond differently to biosecurity threats, this study provides a snapshot of biosecurity practices on Midwestern U.S. swine farms. Further research—both quantitative and qualitative—would help elucidate which specific factors drive variation in how biosecurity is implemented on farms. First, targeted sensitization and practical support that enables producers to identify and implement the components of enhanced biosecurity (SPS-aligned) could reduce assumptions of adequacy and translate intention into practice [7]. Although many respondents were confident about the health status of their herds (Figure 1C; Supplementary Material Table S1), and foreign animal disease (FAD) concerns may prompt stricter measures, without adequate understanding and capacity to apply SPS recommendations the industry would still be at risk should a disease be introduced into the United States. For optimal outreach and engagement, attending veterinarians are key messengers—their opinions were highly regarded—so capacity-building efforts should directly involve them. In addition, while producer confidence in addressing health challenges is encouraging, regular biosecurity gap assessments would help ensure the true status is known and guide where additional effort is needed to strengthen biosecurity.

These data were collected in 2022. Attitudes and routine practice can shift with disease pressure, market conditions, or guidance updates; infrastructure-heavy practices typically change slowly, while frequency-based behaviors may be more responsive. As we did not follow respondents longitudinally, we cannot quantify change since fielding. Estimates are best viewed as a baseline for midwestern operations at that time, and a follow-up survey using comparable items would clarify direction and magnitude of change.

## 4. Limitations

The interpretation of our study results should be done with some considerations in mind. Our study is limited by the number of respondents and the proportional representation of the different production and operation types. Future research should endeavor to collect more diverse data including organic and backyard production systems which may play a pivotal role in cases of disease outbreak. As is common with online administered surveys, we could not ascertain that the respondents filling in the questionnaire were the intended people on the farms. In this study, we intended to involve farm managers/supervisors and veterinarians with intricate knowledge of the daily running of the farms. It is possible that other people within the farm management, like farm owners, filled out some of the questionnaires contributing to either missing data or varied responses to some questions. On participation, this open-link, voluntary survey was distributed through producer networks, so we did not have an enumerated sampling frame or paradata on survey exposure. As a result, we are unable to report a population response rate or to estimate the direction and magnitude of potential coverage or nonresponse bias. Although we collaborated with outreach/extension officers and promoted the survey through events and newsletters to expand reach, we cannot quantify the impact of these strategies on participation. A total of 10 of the 54 respondents provided partial answers to biosecurity-practice items (Q4–Q5). Because this is a descriptive report, we summarized percentages using the full sample (N = 54) for comparability across items, and list item-level counts in Supplementary Table S1. Where nonresponse occurred, reported percentages may underestimate the proportion among those who answered that item. No imputation was performed, and the direction/magnitude of any nonresponse biases are unknown. However, given the level of nonresponse on several practice measures these inferences should be interpreted cautiously. The three-year interval between data collection and this analysis may limit temporal generalizability; post-collection events could alter risk perception and implementation frequency. Without longitudinal data, trends cannot be inferred.

## 5. Conclusions

Consistent, SPS-aligned biosecurity measures are still uneven across the midwestern operations that responded to this study. While many producers felt their current practices were sufficient and expressed confidence in managing disease threats—whether with existing or enhanced measures—what “enhanced biosecurity” actually looks like varied considerably from farm to farm. Building a stronger culture of biosecurity, with ongoing assessments and open reviews of practices, would help address both endemic and foreign disease risks in U.S. swine production. Importantly, producers and workers should play an active role in both evaluating risks and co-designing biosecurity plans, rather than just implementing top-down protocols. Finally, these findings are specific to the respondent group in the Midwest and should not be generalized to all U.S. swine operations. That said, the trends identified here do point to key areas where further, broader investigation could make a real difference.

## Data Availability

Due to confidentiality agreements, anonymized data shall only be made available upon reasonable request to the corresponding author.

## Supplementary Materials

The following supporting information can be downloaded at: https://www.mdpi.com/article/10.3390/ani15172515/s1, Table S1: A summary of survey responses on swine biosecurity among swine producers in the US December 2021 and September 2022.
