# Supplementary material for: Beliefs, Behaviors, and Practices of Farm Biosecurity in the Midwestern U.S. Swine Operations
Source: Animals (Basel). 2025 Aug 27;15(17):2515. doi: 10.3390/ani15172515 (PMC12427511; doi:10.3390/ani15172515)
Supplement: Supplementary file 1 [file animals-15-02515-s001.zip › animals-3806309-supplementary.docx]

Appendix A: A summary of survey responses on swine biosecurity among midwestern swine producers in the US in December 2021 and September 2022.

| **Question** | **Response** | **N = 54** | **Percent** |
| --- | --- | --- | --- |
| Demographics | | | |
| Type of operation | Boar Stud | 1 | 1.9 |
|  | Farrow to Finish | 4 | 7.4 |
|  | Finisher | 3 | 5.6 |
|  | Nursery | 1 | 1.9 |
|  | Nursery, Finisher | 1 | 1.9 |
|  | Sow/Breeding | 2 | 3.7 |
|  | Sow/Breeding, Nursery | 1 | 1.9 |
|  | Sow/Breeding, Wean to Finish | 3 | 5.6 |
|  | Wean to Finish | 5 | 9.3 |
| Type of operation | Contract grower | 4 | 7.4 |
|  | Extensive/outdoor | 3 | 5.6 |
|  | Independent producer | 14 | 25.9 |
| Inventory | 1–99 | 5 | 9.3 |
|  | 100–1999 | 6 | 11.1 |
|  | 2000–4999 | 4 | 7.4 |
|  | Greater than 5000 | 5 | 9.3 |
| Education level attained | College | 12 | 22.2 |
|  | Grad or Professional School | 4 | 7.4 |
|  | High School | 3 | 5.6 |
| Practices | | | |
| Biosecurity manager in place and training plan available | No—there is no specific manager in place, but all employees have received training | 7 | 15.9 |
|  | No—there is no specific training on biosecurity for all | 7 | 15.9 |
|  | Yes—and manager has received training with annual reviews | 3 | 6.8 |
|  | Yes—and manager has received training with less than annual reviews | 1 | 2.3 |
| There is a perimeter buffer area (PBA) | No/Never | 3 | 6.8 |
|  | Sometimes | 2 | 4.5 |
|  | Yes/Always | 7 | 15.9 |
| All entering the PBA entry points follow biosecure entry and exit protocol | No/Never | 2 | 4.5 |
|  | Sometimes | 3 | 6.8 |
|  | Yes/Always | 8 | 18.2 |
| Established cleaning and disinfection points for vehicles and equipment | No/Never | 1 | 2.3 |
|  | Sometimes | 9 | 20.5 |
|  | Yes/Always | 5 | 11.4 |
| Rendering trucks do not enter PBA | No/Never | 13 | 29.5 |
|  | Sometimes | 2 | 4.5 |
|  | Yes/Always | 2 | 4.5 |
| Outside vehicles can enter site | No/Never | 8 | 18.2 |
|  | Sometimes | 1 | 2.3 |
|  | Yes/Always | 3 | 6.8 |
| Is there sharing of equipment with other sites? | No/Never | 4 | 9.1 |
|  | Sometimes | 2 | 4.5 |
|  | Yes/Always | 9 | 20.5 |
| Operational, clearly marked and equipped C&D station at PBA entry points | No/Never | 8 | 18.2 |
|  | Sometimes | 3 | 6.8 |
|  | Yes/Always | 5 | 11.4 |
| Manure disposed in manner that does not expose susceptible animals and follows state and federal regulations | No/Never | 3 | 6.8 |
|  | Sometimes | 3 | 6.8 |
|  | Yes/Always | 9 | 20.5 |
| Feed trucks follow biosecurity hierarchy in their movement | No/Never | 2 | 4.5 |
|  | Sometimes | 4 | 9.1 |
|  | Yes/Always | 11 | 25 |
| Incoming supplies and feed ingredients have evaluation, disinfection, treatment (thermic/chemical) and quarantine period | No/Never | 6 | 13.6 |
|  | Sometimes | 2 | 4.5 |
|  | Yes/Always | 9 | 20.5 |
| Line of separation | No | 3 | 6.8 |
|  | Some sites do | 1 | 2.3 |
|  | Yes | 10 | 22.7 |
| Defined Line of separation | No/Never | 3 | 6.8 |
|  | Sometimes | 2 | 4.5 |
|  | Yes/Always | 7 | 15.9 |
| Line of separation is of size sufficient | No/Never | 1 | 2.3 |
|  | Sometimes | 2 | 4.5 |
|  | Yes/Always | 8 | 18.2 |
| Restricted access across line of separation | No/Never | 1 | 2.3 |
|  | Sometimes | 2 | 4.5 |
|  | Yes/Always | 8 | 18.2 |
| Monitored/recorded movement across line of separation | No/Never | 7 | 15.9 |
|  | Sometimes | 1 | 2.3 |
|  | Yes/Always | 3 | 6.8 |
| Buildings are under lock and key | No | 7 | 15.9 |
|  | Sometimes | 1 | 2.3 |
|  | Yes | 5 | 11.4 |
| Behavioral beliefs | | | |
| Enhanced biosecurity is important and worth implementing | Sometimes | 2 | 3.7 |
|  | Yes/Always | 13 | 24.1 |
| I use biosecurity measures | Sometimes | 3 | 5.6 |
|  | Yes/Always | 12 | 22.2 |
| I use enhanced biosecurity measures | No/Never | 4 | 7.4 |
|  | Not Applicable | 1 | 1.9 |
|  | Sometimes | 3 | 5.6 |
|  | Yes/Always | 7 | 13 |
| I plan to implement enhanced biosecurity measure on my farm | No/Never | 4 | 7.4 |
|  | Not Applicable | 1 | 1.9 |
|  | Sometimes | 3 | 5.6 |
|  | Yes/Always | 7 | 13 |
| Enhanced biosecurity is important for both FAD and endemic diseases | Sometimes | 2 | 3.7 |
|  | Yes/Always | 13 | 24.1 |
| I would implement normal biosecurity measures only if I had a disease problem | No/Never | 8 | 14.8 |
|  | Not Applicable | 4 | 7.4 |
|  | Yes/Always | 3 | 5.6 |
| I would implement enhanced biosecurity measures only if I had a disease problem | No/Never | 6 | 11.1 |
|  | Not Applicable | 3 | 5.6 |
|  | Sometimes | 2 | 3.7 |
|  | Yes/Always | 4 | 7.4 |
| I would implement enhanced biosecurity measures only if we had a FAD in the country | No/Never | 2 | 3.7 |
|  | Not Applicable | 1 | 1.9 |
|  | Sometimes | 6 | 11.1 |
|  | Yes/Always | 6 | 11.1 |
| Implementing enhanced biosecurity measures would improve the welfare of my animals | No/Never | 5 | 9.3 |
|  | Sometimes | 9 | 16.7 |
|  | Yes/Always | 1 | 1.9 |
| Control beliefs | | | |
| Disease outbreaks on my farm are preventable | Not Applicable | 1 | 1.9 |
|  | Sometimes | 11 | 20.4 |
|  | Yes/Always | 2 | 3.7 |
| Once a disease is on my farm I can control it from spreading | No/Never | 1 | 1.9 |
|  | Sometimes | 7 | 13 |
|  | Yes/Always | 6 | 11.1 |
| I know the disease status of my herd | No/Never | 2 | 3.7 |
|  | Sometimes | 1 | 1.9 |
|  | Yes/Always | 11 | 20.4 |
| If I wanted it would be easy for me to implement new biosecurity measures | No/Never | 1 | 1.9 |
|  | Sometimes | 4 | 7.4 |
|  | Yes/Always | 9 | 16.7 |
| Normative beliefs | | | |
| I feel pressure from fellow producers to implement biosecurity measures | No/Never | 10 | 18.5 |
|  | Not Applicable | 1 | 1.9 |
|  | Sometimes | 2 | 3.7 |
|  | Yes/Always | 1 | 1.9 |
| I feel pressure from fellow producers to implement enhanced biosecurity (SPS) measures | No/Never | 5 | 9.3 |
|  | Not Applicable | 2 | 3.7 |
|  | Sometimes | 3 | 5.6 |
|  | Yes/Always | 4 | 7.4 |
| My vet thinks it is important to implement enhanced biosecurity measures | Not Applicable | 5 | 9.3 |
|  | Sometimes | 2 | 3.7 |
|  | Yes/Always | 7 | 13 |
| The opinion of my fellow producers is important regarding implementation of enhanced biosecurity measures | No/Never | 5 | 9.3 |
|  | Not Applicable | 3 | 5.6 |
|  | Sometimes | 2 | 3.7 |
|  | Yes/Always | 4 | 7.4 |
| The opinion of my vet is important regarding implementation of enhanced biosecurity measures | No/Never | 1 | 1.9 |
|  | Not Applicable | 3 | 5.6 |
|  | Sometimes | 1 | 1.9 |
|  | Yes/Always | 9 | 16.7 |
| The opinion of my swine producer association is important regarding implementation of enhanced biosecurity measures | No/Never | 1 | 1.9 |
|  | Not Applicable | 4 | 7.4 |
|  | Sometimes | 3 | 5.6 |
|  | Yes/Always | 6 | 11.1 |
| The opinion of my extension worker is important regarding implementation of enhanced biosecurity measures | No/Never | 2 | 3.7 |
|  | Not Applicable | 1 | 1.9 |
|  | Yes/Always | 1 | 1.9 |
| Other characteristics | | | |
| Are there other animals on the farm | Companion | 2 | 3.7 |
|  | None | 5 | 9.3 |
|  | Production | 3 | 5.6 |
|  | Production, Companion | 5 | 9.3 |
| Do you feed with foreign ingredients? | I do not know the source of all my feed components | 1 | 1.9 |
|  | No | 7 | 13 |
|  | Yes | 6 | 11.1 |
| Outside trucks enter the farm | 2–3x a week | 3 | 5.6 |
|  | Daily | 2 | 3.7 |
|  | Monthly | 2 | 3.7 |
|  | Weekly | 6 | 11.1 |
